# Probing the binding and antiparasitic efficacy of azobenzene G-quadruplex ligands to investigate G4 ligand design†

**DOI:** 10.1039/d4cc03106g

**Published:** 2024-09-13

**Authors:** Javier Ramos-Soriano, Maisie Holbrow-Wilshaw, Eliza Hunt, Y. Jennifer Jiang, Pablo Peñalver, Juan C. Morales, M. Carmen Galan

**Affiliations:** a School of Chemistry, Cantock's Close, University of Bristol BS8 1TS UK; b Instituto de Parasitología y Biomedicina López Neyra, CSIC, PTS Granada Avenida del Conocimiento, 17 18016 Armilla Granada Spain fj.ramos@iiq.csic.es jcmorales@ipb.csic.es m.c.galan@bristol.ac.uk

## Abstract

Novel strategies against parasitic infections are of great importance. Here, we describe a G4 DNA ligand with subnanomolar antiparasitic activity against *T. brucei* and a remarkable selectivity index (IC_50_ MRC-5/*T. brucei*) of 2285-fold. We also correlate the impact of small structural changes to G4 binding activity and antiparasitic activity.

## Abstract 2

Azobenzene ligands targeting G4 DNA exhibit subnanomolar antiparasitic activity against *T. brucei* and a remarkable selectivity index (IC_50_ MRC-5/*T. brucei*) of 2285-fold.

## Graphical abstract

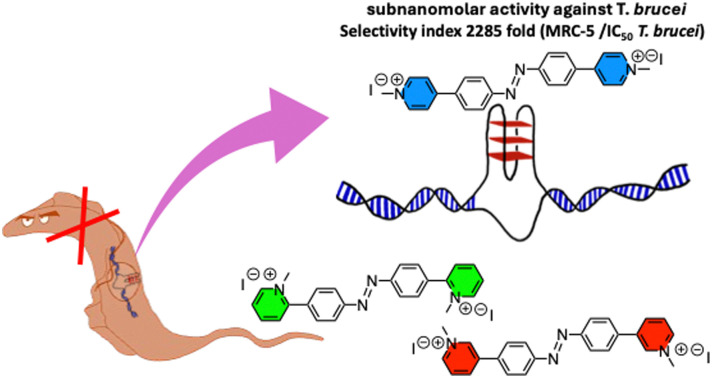

G-quadruplexes (G4s) are nucleic acid secondary structures that form in guanine-rich regions of DNA and RNA in eukaryotes and prokaryotes.^1,2^ G4-sequences have been identified as a potential therapeutic target due to the wide prevalence of quadruplex-forming sequences in human and other genomes (*e.g.* plants, fungi, protozoa, bacteria and viruses), and their involvement in gene regulation and expression.^3–5^

Compared to mammalian systems, studies on protozoan G4s are limited. Early studies reported the presence of human telomeric sequences in addition to several further unique G4s in the genome of protozoan parasites *Trypanosoma brucei* and *Leishmania major.*^6^ More recently, G4-forming sequences have been identified in their genomes *e.g.* EBR1,^7^ which represent a potential new antiparasitic drug target.^7^

African sleeping sickness is a potentially deadly illness caused by the parasite *T. brucei*.^8^ The disease is treatable, but many of the current treatments are old, cause severe side effects^9^ and are becoming increasingly ineffective due to the emergence of drug resistance^10^ and thus there is a need for improved treatments.^11^

Whilst more G4 ligands have been studied as the basis of anticancer and antiviral therapeutics,^5,12–14^ examples of ligands as potential antiparasitic agents have started to emerge.^6,15,16^ Our group and others have identified in recent years G4 ligands based on different scaffolds such as stiff stilbene,^17^ naphthalene diimide,^7,15,18–20^ perylene diimide,^21^ phenanthroline,^22^ quinoxaline^23^ and quinoline cores^24^ and more recently the G4-interacting drug quarfloxin (CX-5461)^25^ and dithienylethenes^26^ with antiplasmodial and antitrypanosomal activity. However, a few structure–activity studies on G4-ligands have prompted the design of G4-targeted small molecules for antiparasitic drug development.^27^ The work herein probes the role of the side chain and the importance of molecular shape, structure and electronics in facilitating G4 binding, and examines whether ligand G4 stabilisation is correlated with antiparasitic activity *in vitro*.

During the course of our studies on the development of novel G-quadruplex ligands to study the role and function of G4 DNA in biology,^12,18^ we became interested in the potential of the azobenzene scaffold to target protozoan G4 DNA. Azobenzene-based ligands have shown favourable G4-binding properties against human telomeric G4 DNA^28–30^ and more recently bacterial G4s.^31^ Additionally, their ease of chemical functionalization makes them ideal candidates for structural tailoring.^32^ To evaluate the effect of the spatial distribution between the aromatic core and the cationic motif towards G4 binding and ultimately antiparasitic activity, three azobenzene scaffolds (1–3, Fig. 1) were examined that had a pyridinium motif with a distinct substitution pattern (2-, 3- or 4-). Pyridinium motifs were chosen as side chains on the basis of our previous results whereby these cationic moieties conferred good G4 affinity.^33,34^

**Fig. 1:**
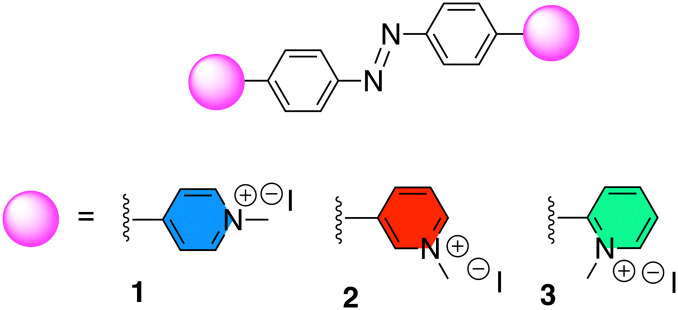
Azobenzene ligands 1–3.

We previously disclosed the synthesis of 4-methyl pyridinium azobenzene 1.^31^ Following a similar synthetic strategy, 3-methyl pyridinium azobenzene derivative 2 was prepared as the bis-iodo salt through a straightforward 2-step procedure (Scheme 1). First, Suzuki coupling of 3-pyridinylboronic acid with 1,2-bis(4-bromophenyl)diazene 4^31^ afforded compound 6 in 81% yield. Next, alkylation with iodomethane provided compound 2 in 97% yield. The synthesis of 2-methyl pyridinium azobenzene 3 (Scheme 1) was more troublesome requiring harsher conditions. As before, it involved the conversion of 2-bromopyridine into the corresponding boronic ester, which was reacted directly with 2-bis(4-bromophenyl)diazene 4^31^ to give 7 in moderate yield. Similarly, alkylation with iodomethane provided 3 in 32% yield, which could be attributed to the low solubility of 7 and steric hindrance. Full synthetic procedures and characterization of the compounds are provided in the ESI.†

**Scheme 1:**
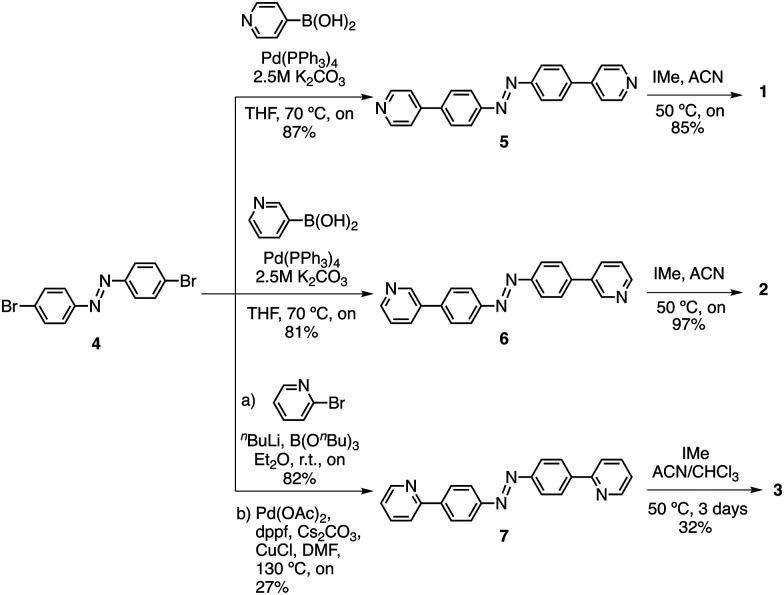
Synthesis of azobenzenes 1–3.

To assess the ligand selectivity for G4 DNA, Förster resonance energy transfer (FRET) melting assays,^35^ which measure ligand-induced stabilisation of the secondary DNA structure as observed by the change in apparent melting temperature (Δ*T*_m_) of the folded species, were conducted at a range of concentrations (1–10 μM) against fluorophore-labelled G4 sequences at 200 nM: polymorphic G4 found in *T. brucei* (Febr1T-K^+^, a mixed G4 topology),^7^ human telomeric G4 in potassium buffer (FhtelT-K^+^, mixed parallel/hybrid G4)^36^ and sodium buffer (FhtelT-Na+, antiparallel G4),^37^ the c-Myc promoter G-quadruplex (FmycT-K^+^, parallel G4)^38^ and a hairpin duplex DNA sequence (F10T-K^+^) (see ESI† for full details). Our results show that 4-Py 1 exhibited higher binding affinity towards G4 sequences when compared to 3-Py 2 and 2-Py 3 (Δ*T*_m_ for 1 was higher by 4 °C and 12 °C than those for compounds 2 and 3, respectively, at 10 μM, Fig. 2 and Table S2, ESI†), with 3 showing minimal stabilization to all DNA sequences. No clear preference towards stabilization of a specific G4 topology was observed for 1 and 2, but with a notable selectivity with respect to duplex DNA since a negligible stabilisation on the duplex DNA model F10T is seen for all compounds at all concentrations tested.

**Fig. 2:**
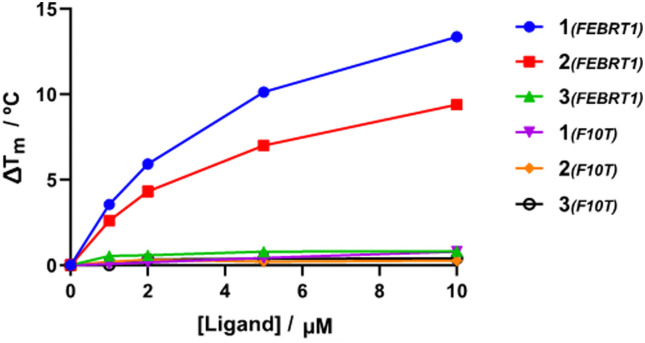
Dependence of Δ*T*_m_ of Febr1T and F10T on the concentration of each ligand.

Having established G4 selectivity for our azobenzene ligands, we then further examined the binding affinity and binding mode under physiologically-relevant conditions of ligands 1–3 with the unlabelled polymorphic EBR1 G4 specific to *T. brucei* in K^+^ buffer, using a combined approach involving ultraviolet-visible absorbance (UV/Vis) and circular dichroism (CD) spectroscopy titration studies.^39^ UV/Vis observed binding isotherms were fitted to an independent-and-equivalent-sites binding model, and the binding constants (*K*_a_) and stoichiometries were determined. Results from the titration of EBR1-K^+^ revealed hypochromicity, and a striking bathochromic shift for 1 and 2 (*ca.* >15 nm) in comparison with the lower red-shift in the absorbance for 3 (*ca.* <10 nm) (Fig. 3A). This effect is indicative of end-stacking ligand binding modes, where the energy of the π–π* transition responsible for the Soret band is lowered by the interactions of the ligand chromophores with the G-tetrad.^40^ The titration with EBR1-K^+^ yielded *K*_a_ values of 0.7 ± 0.05, 0.4 ± 0.04 and 0.02 ± 0.002 × 10^6^ M^−1^ for 1, 2 and 3, respectively (Fig. 3B), whereby 1 shows ≈2- and 35-fold selectivity over ligands 2 and 3. The observed binding isotherms were successfully fitted to a 2 : 1 binding model, which is also in agreement with the potential end-stacking of the ligand on terminal G-tetrads. 2-Py azobenzene 3, which displayed negligible stabilization of EBR1 on FRET, exhibited only subtle perturbations (Fig. 3B), indicative of a weak interaction. Notably, these observed affinities mirror the trends observed in the thermal melting assay, with 4-Py azobenzene 1 emerging as the most potent G4 ligand of the series.

**Fig. 3:**
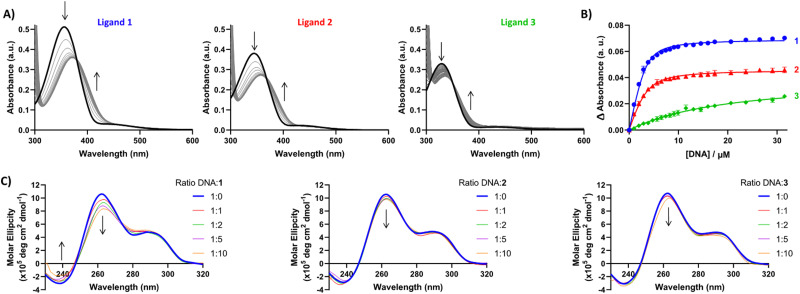
(A) UV-Vis spectra of ligands 1–3 titrated with EBR1-K^+^. (B) UV-Vis binding isotherm for the association between ligands 1, 2 and 3 with EBR1, following the change in ligand absorbance at 420, 405 and 390 nm, respectively. Binding constants fitted using an independent-and-equivalent-sites binding model, with 2 : 1 ligand : DNA stoichiometry. Ligand concentration was 10 μM, with oligonucleotide concentration varied up to 30 μM. (C) CD spectra of ligands 1–3 titrated with EBR1-K^+^. Oligonucleotide concentration was 5 μM, with ligand concentration varied up to 10 equivalents (50 μM). No CD was observed for the free ligands in the absence of G4 sequence (data not shown).

These results suggest that the spatial positioning of the pyridinium N is key for the inherent selectivity observed towards four-stranded structures over duplex sequences and it is also crucial for optimal binding with 4-Py 1 and 3-Py 2 exhibiting micromolar G4 affinity, whilst 2-Py displays affinity 1 order of magnitude lower. The lack of G4 stabilization by 3 might be attributed to the shorter distance between the N atoms of both pyridinium rings, which do not facilitate the correct orientation for G4 groove binding.

To further probe the different G4 stabilization modes and potential topology changes induced by 1–3, circular dichroism (CD) experiments were also conducted on EBR1-K^+^. The CD spectrum of EBR1 is characterized by two positive bands at 260 and 295 nm and a negative band at 240 nm indicative of a predominant parallel G-quadruplex topology.^7^ Although no conformational change was observed in K^+^ conditions (Fig. 3C), binding of all ligands with the EBR1 sequence is evidenced by perturbation of the positive (260 nm) and negative (240 nm) bands. The effect is most striking for azobenzene 1, which is consistent with this compound being the more potent of the three pyridinium ligands investigated in the current study. Indeed, 1 induces hypochromicity in the positive band at 260 nm and the negative band at 240 nm. These effects suggest that the ligand induces a disruption of the folded topology, possibly arising from an intercalative binding mechanism at higher concentration. Lesser spectral perturbations were observed for azobenzene 3, corroborating the results from the FRET and UV-Vis assays, where weaker stabilization of G4 was observed over the range of concentrations studied.

Next, we examined the cytotoxicity and antiparasitic activity of ligands 1–3 against *T*. *brucei* and *L. major* strains and MRC5 fibroblast cells as a healthy control (Table 1). Interestingly, 4-Py azobenzene 1 shows submicromolar efficiency against *L. major* and subnanomolar efficiency against *T. brucei*. In fact, the antitrypanosomal activity observed within the series follows the same tendency observed in binding to quadruplexes, with 1 being the most efficient, then 2 and finally 3 with the lowest activity. Remarkably, the selectivity index (IC_50_ MRC-5/IC_50_*T. brucei*) was 2285 fold in the range of that obtained for suramin.

**Table 1:** IC_50_ values in μM measured for MRC-5 and *T. brucei*, together with the control drug suramin. Data in bold corresponds to the best antiparasitic activity

Ligand	MRC5	*L. major*	*T. brucei*	SI MRC5/*L. major*	SI MRC5/T. *brucei*
1	1.6 ± 0.5	0.7 ± 0.2	**0.0007 ± 0.00008**	2.3	**2285.7**
2	>100	10.5 ± 1	0.37 ± 0.10	>9.5	>270
3	30.5 ± 9.8	53.7 ± 4.7	18.8 ± 0.47	<1	1.6
Suramin	350	—	0.038 ± 0.003	—	9210

In conclusion, we describe three G-quadruplex ligands based on an azobenzene scaffold featuring methyl pyridinium side chains with 2-, 3- or 4-substitution pattern with regards to the azobenzene core, which varies the overall spatial presentation of the cationic head. Our study reveals that although the structural changes are relatively small, a significant effect is seen on G4 binding affinity as demonstrated by FRET, UV-Vis and CD experiments. We found that 4-Py 1 exhibited higher binding affinity and selectivity towards G4 sequences of mixed topology (*e.g.* Febr1T-K^+^ and F21T-K^+^) when compared to 3-Py 2 and 2-Py 3, with 3, which features the *N*-methyl group closer to the azobenzene core, showing minimal stabilization of all DNA sequences. These results suggest that the position of the positively charged N in the pyridinium ring is a key driving force for G4 stabilization and selectivity, and should be considered as an important factor when designing or tuning G4 interactive compounds. Furthermore, we were able to correlate G4 binding affinity with antiparasitic activity and found that 4-Py azobenzene 1 exhibited submicromolar efficiency against *L. major* and subnanomolar efficiency against *T. brucei* and a superb selectivity index against MRC5 fibroblast cells. Although there is no preference for specific G4 topologies when we compare all the topologies screened, the ligand is very selective towards G4 over duplex DNA. The observed antiparasitic activity and selectivity index may come from a variety of reasons, such as differences in cellular uptake between the parasites and mammalian cells, differences in cell cycle rate (human typical cell cycle is 24 h, whereas the *T. brucei* divides every 2 h) or differential nucleus entry due to the dissimilar nuclear membrane composition.^41^ Our study provides insights into key structural features required for G4 binding and target selectivity and paves the way for the development of novel antiparasitic strategies.

MCG thanks ERC-COG: 648239, J. R.-S. thanks MSCA fellowship (project 843720-BioNanoProbes). E. H. thanks EPSRC EP/L015366/1 and J. Y. J. (EPSRC EP/L015366/1 and EP/S026215/1) for their PhD studentships. JCM thanks the Spanish Ministerio de Ciencia, Innovación y Universidades (Grant PID2021-127109OB-I00).

## Data availability

The data supporting this article have been included as part of the ESI.† This includes synthetic protocols and characterization data for all compounds, all biophysical characterization using FRET, CD and UV-Vis and details on antiparasitic assays.

## Conflicts of interest

There are no conflicts to declare.

## Supplementary Material

CC-060-D4CC03106G-s001

## References

[cit1] Saranathan N., Vivekanandan P. (2019). Trends Microbiol..

[cit2] Gellert M., Lipsett M. N., Davies D. R. (1962). Proc. Natl. Acad. Sci. U. S. A..

[cit3] Alberti P., Mergny J.-L. (2003). Proc. Natl. Acad. Sci. U. S. A..

[cit4] Alberti P., Bourdoncle A., Saccà B., Lacroix L., Mergny J.-L. (2006). Org. Biomol. Chem..

[cit5] Neidle S. (2016). J. Med. Chem..

[cit6] Blackburn E. H., Challoner P. B. (1984). Cell.

[cit7] Belmonte-Reche E., Martínez-García M., Guédin A., Zuffo M., Arévalo-Ruiz M., Doria F., Campos-Salinas J., Maynadier M., López-Rubio J. J., Freccero M., Mergny J.-L., Pérez-Victoria J. M., Morales J. C. (2018). J. Med. Chem..

[cit8] Franco J. R., Cecchi G., Priotto G., Paone M., Diarra A., Grout L., Mattioli R. C., Argaw D. (2017). PLoS Neglected Trop. Dis..

[cit9] Luscher A., de Koning H. P., Maser P. (2007). Curr. Pharm. Des..

[cit10] Baker N., de Koning H. P., Maser P., Horn D. (2013). Trends Parasitol..

[cit11] Field M. C., Horn D., Fairlamb A. H., Ferguson M. A. J., Gray D. W., Read K. D., De Rycker M., Torrie L. S., Wyatt P. G., Wyllie S., Gilbert I. H. (2017). Nat. Rev. Microbiol..

[cit12] Ramos-Soriano J., Galan M. C. (2021). JACS Au.

[cit13] Balasubramanian S., Hurley L. H., Neidle S. (2011). Nat. Rev. Drug Discovery.

[cit14] Nadai M., Doria F., Frasson I., Perrone R., Pirota V., Bergamaschi G., Freccero M., Richter S. N. (2024). ACS Infect. Dis..

[cit15] Zuffo M., Stucchi A., Campos-Salinas J., Cabello-Donayre M., Martinez-Garcia M., Belmonte-Reche E., Perez-Victoria J. M., Mergny J. L., Freccero M., Morales J. C., Doria F. (2019). Eur. J. Med. Chem..

[cit16] Monti L., Antonio M. D. (2023). ChemBioChem.

[cit17] O'Hagan M. P., Peñalver P., Gibson R. S. L., Morales J. C., Galan M. C. (2020). Chem. – Eur. J..

[cit18] Perez-Soto M., Penalver P., Street S. T. G., Weenink D., O'Hagan M. P., Ramos-Soriano J., Jiang Y. J., Hollingworth G. J., Galan M. C., Morales J. C. (2022). Bioorg. Med. Chem..

[cit19] Arevalo-Ruiz M., Doria F., Belmonte-Reche E., De Rache A., Campos-Salinas J., Lucas R., Falomir E., Carda M., Perez-Victoria J. M., Mergny J. L., Freccero M., Morales J. C. (2017). Chem. – Eur. J..

[cit20] Belmonte-Reche E., Benassi A., Penalver P., Cucchiarini A., Guedin A., Mergny J. L., Rosu F., Gabelica V., Freccero M., Doria F., Morales J. C. (2022). Eur. J. Med. Chem..

[cit21] Street S. T. G., Peñalver P., O'Hagan M. P., Hollingworth G. J., Morales J. C., Galan M. C. (2021). Chem. Eur. J..

[cit22] Guillon J., Cohen A., Boudot C., Monic S., Savrimoutou S., Moreau S., Albenque-Rubio S., Lafon-Schmaltz C., Dassonville-Klimpt A., Mergny J. L., Ronga L., Bernabeu de Maria M., Lamarche J., Lago C. D., Largy E., Gabelica V., Moukha S., Dozolme P., Agnamey P., Azas N., Mullie C., Courtioux B., Sonnet P. (2022). Pathogens.

[cit23] Guillon J., Cohen A., Gueddouda N. M., Das R. N., Moreau S., Ronga L., Savrimoutou S., Basmaciyan L., Monnier A., Monget M., Rubio S., Garnerin T., Azas N., Mergny J. L., Mullie C., Sonnet P. (2017). J. Enzyme Inhib. Med. Chem..

[cit24] Guillon J., Cohen A., Boudot C., Valle A., Milano V., Das R. N., Guedin A., Moreau S., Ronga L., Savrimoutou S., Demourgues M., Reviriego E., Rubio S., Ferriez S., Agnamey P., Pauc C., Moukha S., Dozolme P., Nascimento S. D., Laumaille P., Bouchut A., Azas N., Mergny J. L., Mullie C., Sonnet P., Courtioux B. (2020). J. Enzyme Inhib. Med. Chem..

[cit25] Craven H. M., Nettesheim G., Cicuta P., Blagborough A. M., Merrick C. J. (2023). Int. J. Parasitol.: Drugs Drug Resist..

[cit26] O'Hagan M. P., Ramos-Soriano J., Haldar S., Sheikh S., Morales J. C., Mulholland A. J., Galan M. C. (2020). Chem. Commun..

[cit27] Pérez-Soto M., Ramos-Soriano J., Peñalver P., Belmonte-Reche E., O’Hagan M. P., Cucchiarini A., Mergny J. L., Galán M. C., López-López M. C., Thomas M. C., Morales J. C. (2024). Eur. J. Med. Chem..

[cit28] Wang X., Huang J., Zhou Y., Yan S., Weng X., Wu X., Deng M., Zhou X. (2010). Angew. Chem., Int. Ed..

[cit29] Xing X., Wang X., Xu L., Tai Y., Dai L., Zheng X., Mao W., Xu X., Zhou X. (2011). Org. Biomol. Chem..

[cit30] Tian T., Song Y., Wang J., Fu B., He Z., Xu X., Li A., Zhou X., Wang S., Zhou X. (2016). J. Am. Chem. Soc..

[cit31] Takebayashi Y. , Ramos-Soriano J., Jiang Y. J., Samphire J., Belmonte-Reche E., O’Hagan M. P., Gurr C., Heesom K. J., Lewis P. A., Samernate T., Nonejuie P., Spencer J. and Galan M. C., *bioRxiv*, 2024, preprint 10.1101/2022.09.01.506212

[cit32] Jerca F. A., Jerca V. V., Hoogenboom R. (2022). Nat. Rev. Chem..

[cit33] O'Hagan M. P., Haldar S., Duchi M., Oliver T. A. A., Mulholland A. J., Morales J. C., Galan M. C. (2019). Angew. Chem., Int. Ed..

[cit34] O'Hagan M. P., Haldar S., Morales J. C., Mulholland A. J., Galan M. C. (2021). Chem. Sci..

[cit35] De Cian A., Guittat L., Kaiser M., Saccà B., Amrane S., Bourdoncle A., Alberti P., Teulade-Fichou M.-P., Lacroix L., Mergny J.-L. (2007). Methods.

[cit36] Luu K. N., Phan A. T., Kuryavyi V., Lacroix L., Patel D. J. (2006). J. Am. Chem. Soc..

[cit37] Wang Y., Patel D. J. (1993). Structure.

[cit38] Ambrus A., Chen D., Dai J., Jones R. A., Yang D. (2005). Biochemistry.

[cit39] Nicoludis J. M., Barrett S. P., Mergny J. L., Yatsunyk L. A. (2012). Nucleic Acids Res..

[cit40] Falanga A. P., D'Urso A., Travagliante G., Gangemi C. M. A., Marzano M., D'Errico S., Terracciano M., Greco F., De Stefano L., Dardano P., Rea I., Piccialli G., Oliviero G., Borbone N. (2024). Int. J. Biol. Macromol..

[cit41] Daniels J. P., Gull K., Wickstead B. (2010). Cell biology of the trypanosome genome. Microbiol. Mol. Biol. Rev..

