# Supplementary material for: Probing the binding and antiparasitic efficacy of azobenzene G-quadruplex ligands to investigate G4 ligand design
Source: Chem Commun (Camb). 2024 Sep 13;60(81):11520–3. doi: 10.1039/d4cc03106g (PMC11418008; doi:10.1039/d4cc03106g)
Supplement: CC-060-D4CC03106G-s001 [file CC-060-D4CC03106G-s001.pdf]

Supplementary Information (SI) for ChemComm.
This journal is © The Royal Society of Chemistry 2024

Probing the binding and antiparasitic efficacy of azobenzene G-quadruplex ligands to investigate G4 ligand design principles

Javier Ramos-Soriano[1,2]*, Maisie Holbrow-Wilshaw[1], Eliza Hunt[1], Y. Jennifer Jiang[1], Pablo Peñalver[3], Juan C. Morales[3]*, M. Carmen Galan[1]*

[a]School of Chemistry, Cantock's Close, BS8 1TS, University of Bristol, UK; [b]Present address: Instituto de Investigaciones Químicas (IIQ), CSIC – Universidad de Sevilla, Av. Américo Vespucio 49, Seville, 41092, Spain; [c]Instituto de Parasitología y Biomedicina López Neyra, CSIC, PTS Granada, Avenida del Conocimiento, 17, 18016, Armilla, Granada, Spain.

†Corresponding authors: fj.ramos@iiq.csic.es, jcmorales@ipb.csic.es and m.c.galan@bristol.ac.uk

1. Synthesis and characterization *S1*
2. FRET melting assays *S7*
3. Determination of apparent dissociation constants by UV-Visible spectroscopy *S9*
4. Circular dichroism titrations *S10*
5. Biological activity *S11*
6. References *S12*

1. Synthesis and characterization.

Reagents and solvents were purchased as reagent grade and used without further purification. Reactions requiring anhydrous conditions were performed under N_2; glassware and needles were either flame dried immediately prior to use, or placed in an oven (150 °C) for at least 2 h and allowed to cool in a desiccator or under reduced pressure. (*E*)-4,4'-dibromoazobenzene (**4**) was prepared according to previously reported procedure.[1] For column chromatography, silica gel 60 (230-400 mesh, 0.040-0.063 mm) was purchased from E. Merck. Thin Layer Chromatography (TLC) was performed on aluminium sheets coated with silica gel 60 F_{254} purchased from E. Merck, visualization by UV light. NMR spectra were recorded on a Bruker AC 400 or AC500 with solvent peaks as reference. ^{1}H and ^{13}C NMR spectra were obtained for solutions in $CDCl_3$ and DMSO-d_6. All the assignments were confirmed by one- and two-dimensional NMR experiments (DEPT, COSY, HSQC and HMBC). Mass spectra were obtained by the University of Bristol mass spectrometry service by electrospray ionisation (ESI).

(*E*)-4,4'-bis(pyridin-3-yl)azobenzene (6)

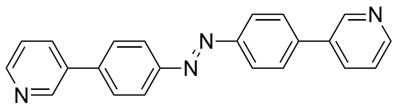

A suspension of (*E*)-4,4'-dibromoazobenzene (**4**) (250 mg, 0.735 mmol), $Pd(PPh_3)_4$ (85.0 mg, 74.0 μmol), ethyleneglycol (1 drop) and 3-pyridinylboronic acid (271 mg, 2.21 mmol) in a mixture of THF (10 mL) and aq. 2.5 M K_2CO_3 (2.5 mL) was bubbled with N_2 for 10 min. The resulting solution was heated at 70 °C overnight. After cooling to room temperature, water was added. The aqueous layer was extracted with DCM (x2) and the combined organic extractions dried over $MgSO_4$, filtered and concentrated *in vacuo*. The residue was purified by flash silica chromatography (DCM/MeOH, 40:1), afforded compound **6** (200 mg, 81%) as an orange amorphous solid. ^{1}H NMR (400 MHz, $CDCl_3$) δ: 8.92 (dd, J = 2.4, 0.9 Hz, 2H), 8.63 (dd, J = 4.8, 1.6 Hz, 2H), 8.04 (d, J = 7.7 Hz, 4H), 7.92 (ddd, J = 7.9, 2.4, 1.6 Hz, 2H), 7.73 (d, J = 7.7 Hz, 4H), 7.38 (ddd, J = 7.9, 4.8, 0.9 Hz, 2H); ^{13}C NMR (101 MHz, $CDCl_3$) δ: 152.3, 149.2, 148.4, 140.5, 135.7, 134.4, 127.9, 123.8, 123.7; ESI-HRMS for $C_{22}H_{17}N_4$ $[M+H]^+$ calcd: 337.1448, found: 337.1455.

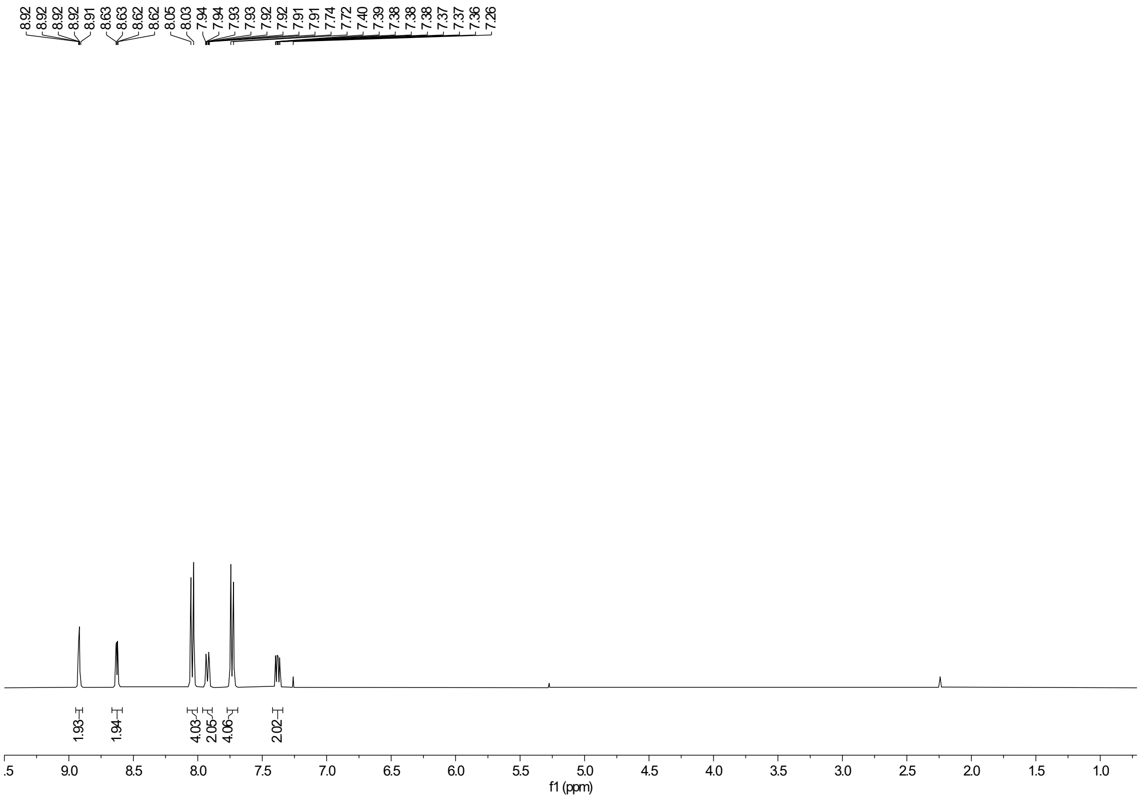

^{1}H NMR spectrum of compound **6** ($CDCl_3$, 400 MHz).

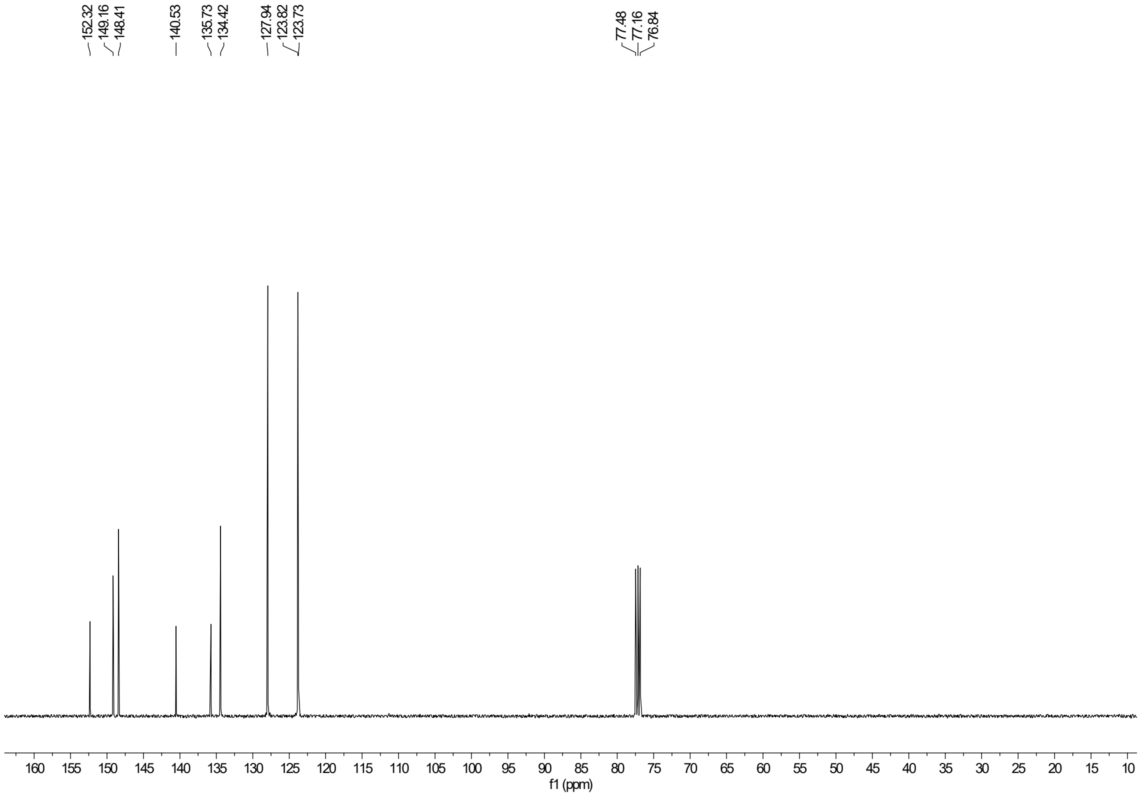

^{13}C NMR spectrum of compound **6** ($CDCl_3$, 101 MHz).

Compound 2.

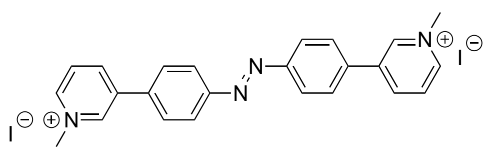

To a solution of compound **6** (100 mg, 0.30 mmol) in acetonitrile (8 mL), methyl iodide (148 µL, 2.38 mmol) was added. The solution was stirred in a sealed vessel at 50 °C overnight. The generated solid was filtered and washed with ether. Following filtration, compound **2** (180 mg, 97%) was obtained as an orange powder. ^{1}H NMR (400 MHz, DMSO-d_6) δ: 9.56 (s, 2H), 9.02 (t, J = 7.2 Hz, 4H), 8.26 (dd, J = 8.2, 6.0 Hz, 2H), 8.20 (d, J = 8.8 Hz, 4H), 8.15 (d, J = 8.7 Hz, 4H), 4.47 (s, 6H); ^{13}C NMR (101 MHz, DMSO-d_6) δ: 152.4, 144.3, 144.0, 142.4, 137.8, 136.1, 128.8, 127.7, 123.7, 48.2; ESI-HRMS for $C_{24}H_{22}IN_4^+$ [M-I]$^+$ calcd: 493.0884, found: 493.0893.

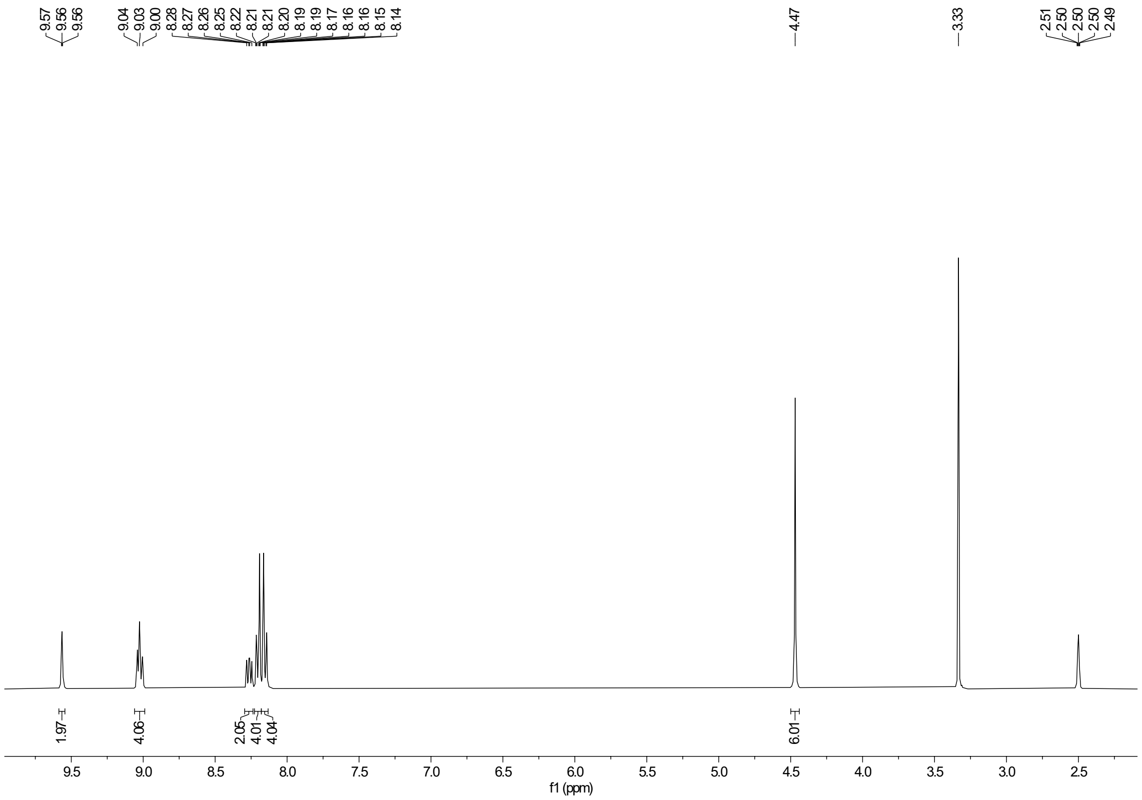

^{1}H NMR spectrum of compound **2** (DMSO-d_6, 400 MHz).

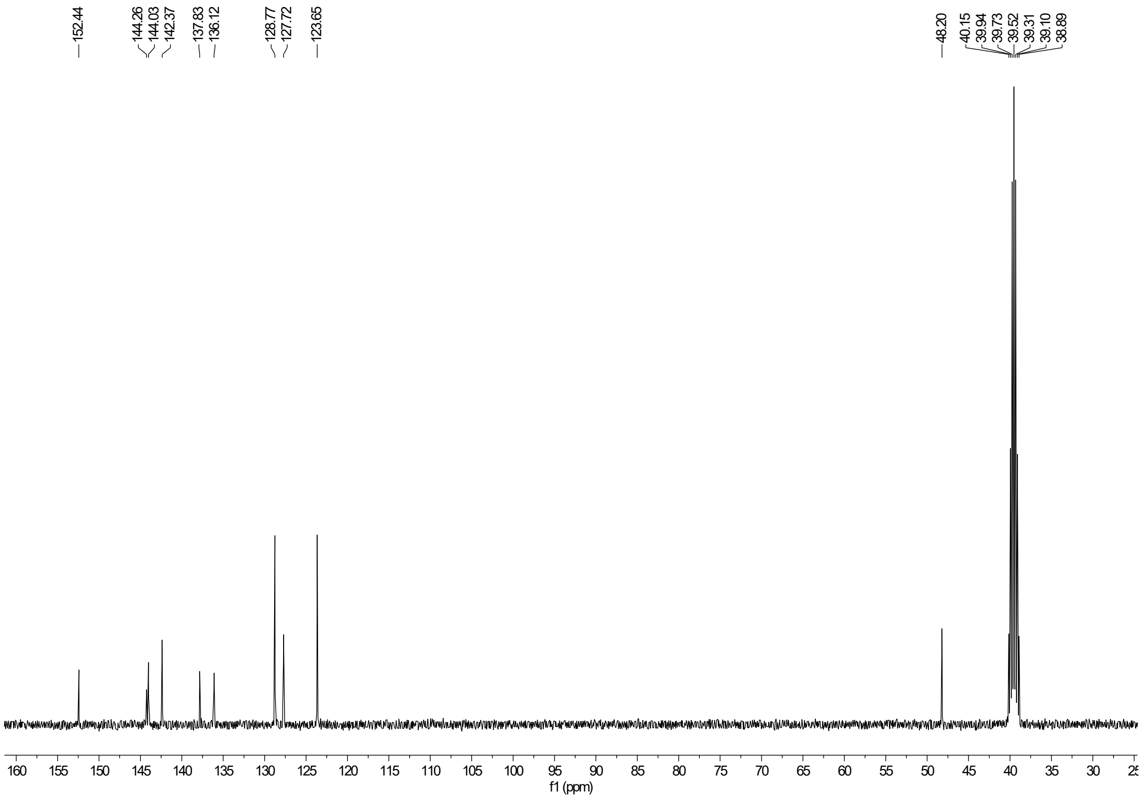

^{13}C NMR spectrum of compound **2** (DMSO-d_6, 101 MHz).

(*E*)-4,4'-bis(pyridin-2-yl)azobenzene (7)

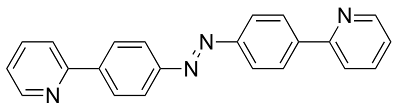

To a solution of 2-bromopyridine (300 mg, 1.90 mmol) in dry Et_2O (10 mL), previously cooled to -78 °C and under an N_2 atmosphere, ^{n}BuLi (2.5 M, 835 µL) was added dropwise and the reaction stirred at this temperature for 1 hour. $B(O^nBu)_3$ (768 µL, 2.85 mmol) was added dropwise and the temperature was raised to r.t. naturally overnight. After that, the reaction was concentrated *in vacuo* to afford the corresponding boronate ester (457 mg, 82%), which was used directly in the next step without further purification. A suspension of (*E*)-4,4'-dibromoazobenzene (**4**) (200 mg, 0.588 mmol), Cs_2CO_3 (3.83 g, 11.80 mmol), CuCl (291 mg, 2.94 mmol), $Pd(OAc)_2$ (52.8 mg, 0.23 mmol), dppf (260 mg, 0.47 mmol) and the above prepared boronate ester (341 mg, 1.23 mmol) in dry DMF (25 mL) was bubbled with N_2 for 10 min. The resulting solution was heated at 130 °C overnight. After cooling to room temperature, the mixture was diluted with CH_2Cl_2 and washed with water (x3). The organic layer was dried over $MgSO_4$, filtered and concentrated *in vacuo*. The residue was purified by flash silica chromatography (DCM/MeOH 100:1 → 50:1), afforded compound **7** (53.0 mg, 27%) an orange amorphous solid. ^{1}H NMR (500 MHz, $CDCl_3$) δ: 8.75 (s, 2H), 8.19 (d, J = 8.1 Hz, 4H), 8.07 (d, J = 8.0 Hz, 4H), 7.86 – 7.77 (m, 4H), 7.29 (t, J

= 6.1 Hz, 2H); ^{13}C NMR (126 MHz, $CDCl_3$) δ: 156.6, 153.1, 150.0, 141.9, 137.0, 127.8, 123.6, 122.8, 121.0; ESI-HRMS for $C_{22}H_{17}N_4$ $[M+H]^+$ calcd: 337.1448, found: 337.1446.

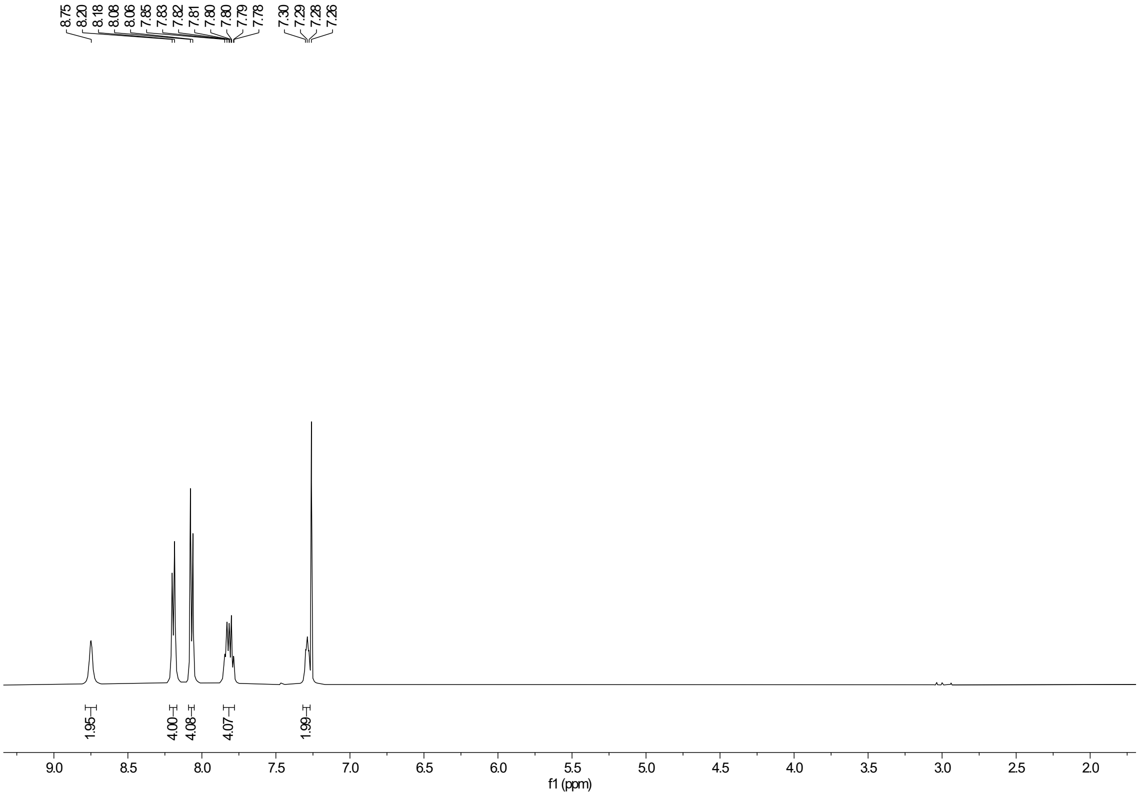

^{1}H NMR spectrum of compound **7** ($CDCl_3$, 500 MHz).

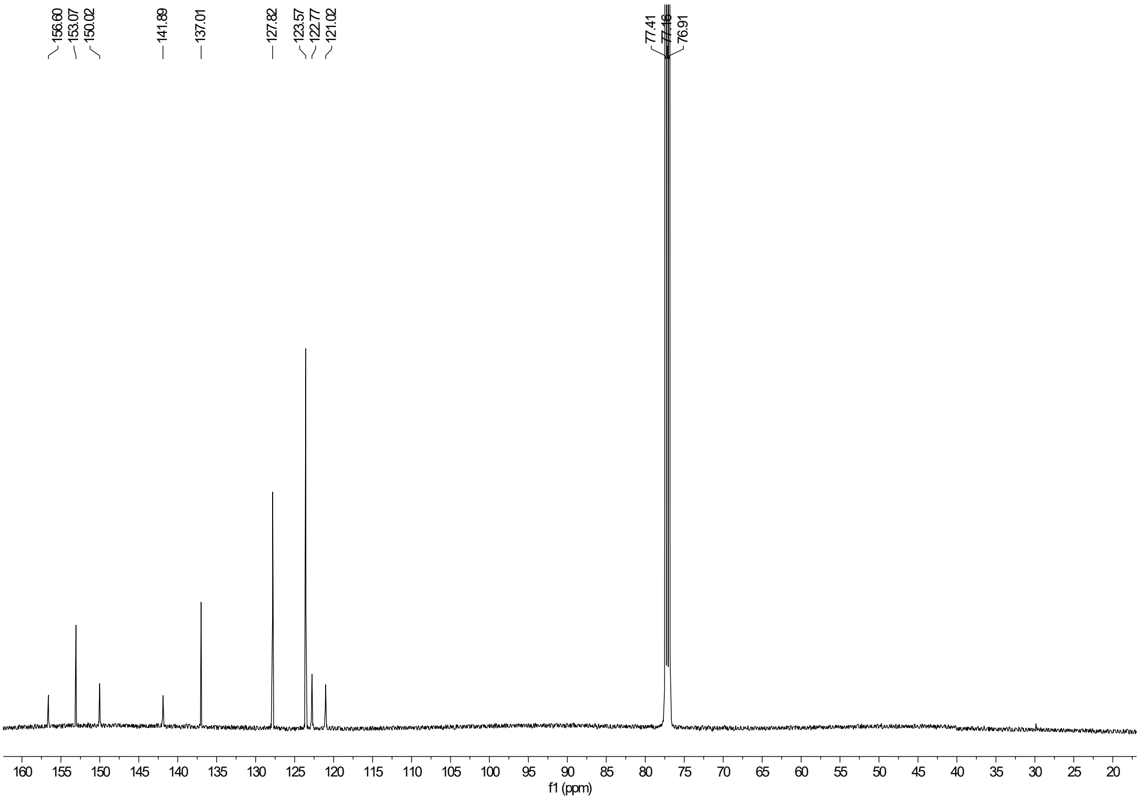

^{13}C NMR spectrum of compound **7** ($CDCl_3$, 126 MHz).

Compound 3.

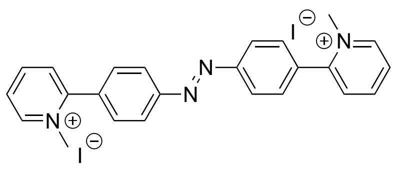

To a solution of compound **7** (50 mg, 0.15 mmol) in a mixture of ACN/$CHCl_3$ (1:1, 6 mL), methyl iodide (296 µL, 4.76 mmol) was added. The solution was stirred in a sealed vessel at 50 °C for 3 days. The generated solid was filtered and washed with ACN. Following filtration, compound **3** (32 mg, 32%) was obtained as an orange powder. ^{1}H NMR (500 MHz, DMSO-d_6) δ: 9.18 (dd, J = 6.3, 1.4 Hz, 2H), 8.69 (td, J = 7.9, 1.4 Hz, 2H), 8.25 – 8.15 (m, 8H), 7.96 (d, J = 8.5 Hz, 4H), 4.19 (s, 6H); ^{13}C(APT) NMR (126 MHz, DMSO-d_6) δ: 154.1, 152.9, 147.0, 145.6, 134.8, 131.0, 129.9, 127.1, 123.2, 47.3; ESI-HRMS for $C_{24}H_{22}IN_4^+$ $[M-I]^+$ calcd: 493.0884, found 493.0811.

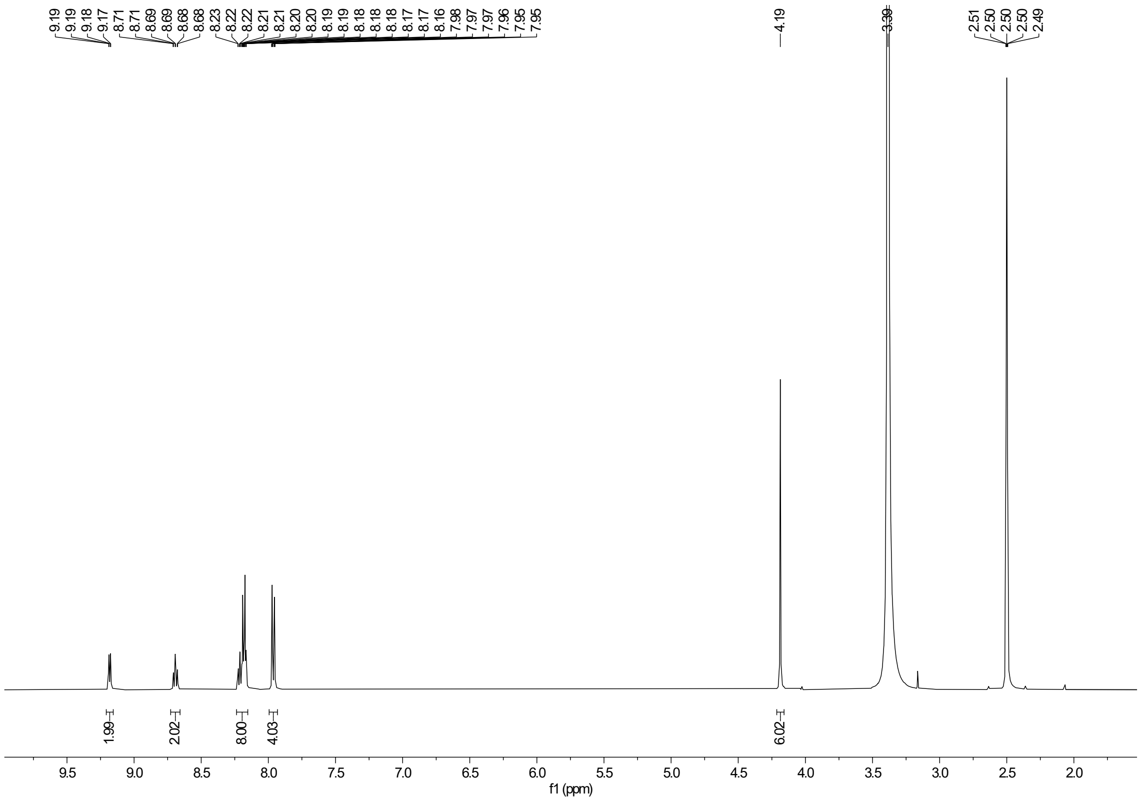

^{1}H NMR spectrum of compound **3** (DMSO-d_6, 500 MHz).

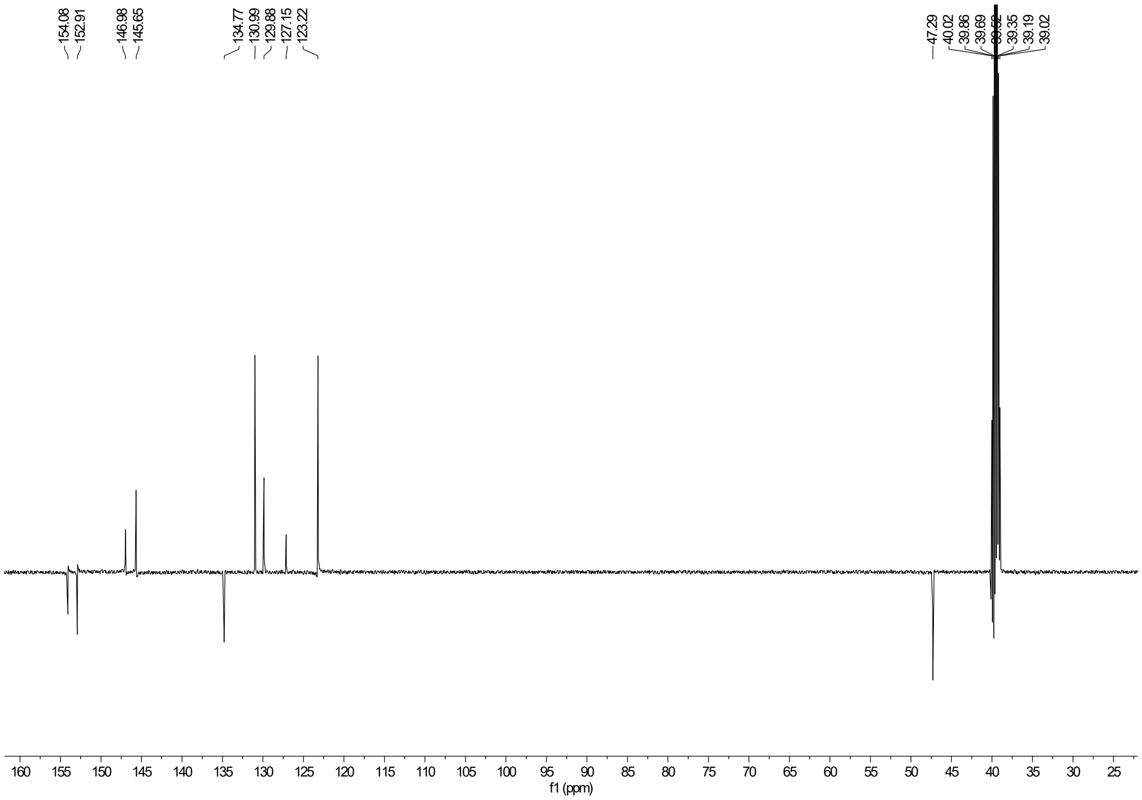

^{13}C (APT) NMR spectrum of compound **3** (DMSO-d_6, 126 MHz).

2. FRET melting assays

Fluorescence resonance energy transfer (FRET) melting assays were performed according to the procedure reported by De Cian and co-workers[2] on Roche LightCycler 480 qPCR instrument. In these assays, oligonucleotides of interest were obtained labelled at the 5' and 3' ends with FAM (a fluorescence donor) and TAMRA (a fluorescence quencher), respectively. In the folded state, proximity of the donor and quencher result in no observed fluorescence from FAM, since energy is transferred non-radiatively to TAMRA by FRET. As the temperature is raised and the secondary structure denatures, the fluorophores move further apart and hence the fluorescence signal increases. From the resulting curve, the characteristic melting temperature ($T_{1/2}$) is defined as that at which the normalised fluorescence signal equals 0.5. The change in melting temperature (ΔT_m) induced by a small molecule ligand compared to that of the oligonucleotide in the absence of ligand provides an indication of the ligand's ability to stabilise the G4 structure. The assay is shown in schematic form below:

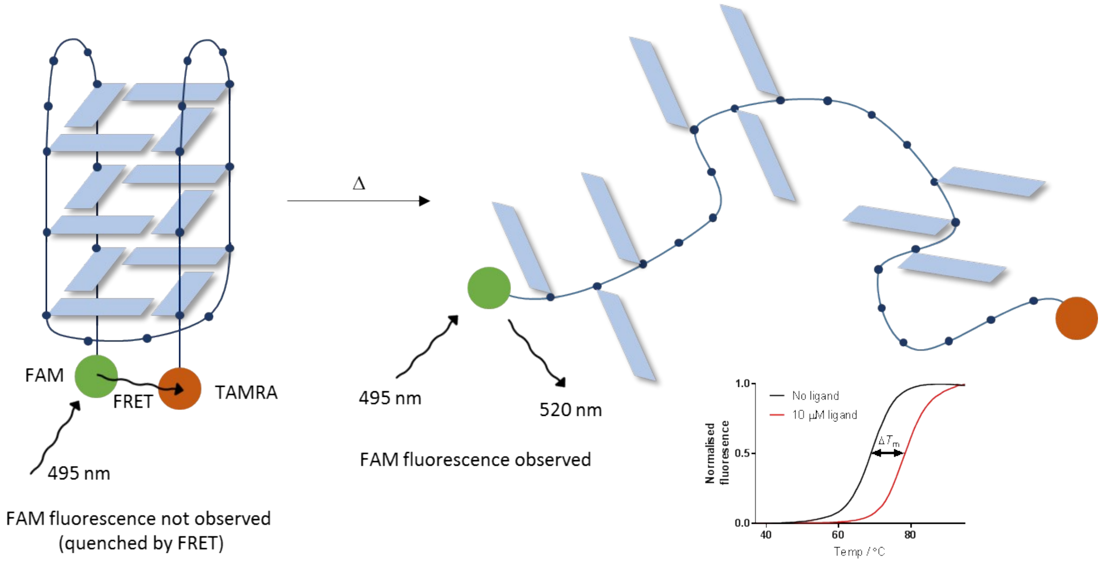

The method consisted of holding at 25 °C for 5 min, before heating at 1 °C/min to 96 °C in 1 °C increments, followed by monitoring the fluorescence output at each increment for 1 min. The fluorescence emission of FAM was followed at 516 nm, with a 10 nm full width at half-maximum filter and an 8-fold gain, after excitation at 492 nm with a 9 nm full width at half-maximum filter. All oligonucleotides used were purchased from Eurogentec (Belgium), purified by HPLC and delivered dry (Table S1). Oligonucleotide concentrations were determined by UV-absorbance using a NanoDrop 2000 Spectrophotometer from Thermo Scientific. All sequences were annealed before use by heating for 2 minutes at 90°C and then placed immediately into ice. The final concentration of oligonucleotide was 200 nM in all cases. The buffer used depended on the sequence in question: 10 mM KCl, 90 mM LiCl and 10 mM Li cacodylate for Febr1T-K^+, FhtelT-K^+ and F10T-K^+; 100 mM NaCl and 10 mM Li cacodylate for FhtelT-Na^+; and 1 mM KCl, 99 mM LiCl and 10 mM Li cacodylate for FmycT-K^+. Ligand concentrations were either 1 μM, 2 μM, 5 μM or 10 μM. Each sample was tested in duplicate on the same plate, and each plate was repeated in at least duplicate to assess the reproducibility of all results.

Appropriate control experiments were also carried out for each sample set. Data processing was carried out using Origin 9, with $\Delta T_{1/2}$ used to represent ΔT_m.

Table S1. Oligonucleotides employed in FRET melting assays

DNA model	Sequence
**Febr1T (T.brucei G4)**	5’-FAM-GGGCAGGGGGTGATGGGGAGGAGCCAGGG-TAMRA-3’
**FhtelT (human telomeric G4)**	5’-FAM-GGGTTAGGGTTAGGGTTAGGG-TAMRA-3’
**FmycT (c-myc promoter G4)**	5’-FAM-TTGAGGGTGGGTAGGGTGGGTAA-TAMRA-3’
**F10T (duplex)**	5’-FAM-TATAGCTATA-HEG-TATAGCTATA-TAMRA-3’

FAM = 6-carboxyfluorescein; TAMRA = 6-carboxy-tetramethylrhodamine; HEG = $[(-CH_2CH_2O-)_6$

Table S2. DNA stabilisation of compounds **1-3** assessed via FRET Melting Assay at 1, 2, 5 and 10 µM ligand and 200 nM DNA concentration. Buffers composition was 10 mM KCl, 90 mM LiCl and 10 mM Li cacodylate for Febr1T-K^+, FhtelT-K^+ and F10T-K^+; 100 mM NaCl and 10 mM Li cacodylate for FhtelT-Na^+; and 1 mM KCl, 99 mM LiCl and 10 mM Li cacodylate for FmycT-K^+.

[Ligand]	1 µM	2 µM	5 µM	10 µM
FEBR1T-K $^+$		ΔT_m (ºC)		
**1**	3.6 ± 0.2	5.9 ± 0.2	10.1 ± 0.2	13.4 ± 0.2
**2**	2.6 ± 0.2	4.3 ± 0.3	7.0 ± 0.2	9.4 ± 0.3
**3**	0.5 ± 0.1	0.6 ± 0.2	0.8 ± 0.1	0.8 ± 0.2
FhtelT-K $^+$				
**1**	4.6 ± 0.3	7.1 ± 0.3	10.8 ± 0.3	14.0 ± 0.6
**2**	2.5 ± 0.2	4.9 ± 0.3	7.6 ± 0.3	10.7 ± 0.4
**3**	0.6 ± 0.3	0.7 ± 0.3	1.1 ± 0.5	1.5 ± 0.3
FhtelT-Na $^+$				
**1**	1.4 ± 0.4	2.3 ± 0.3	6.5 ± 0.4	10.3 ± 0.9
**2**	0.2 ± 0.04	2.0 ± 0.4	3.9 ± 0.3	6.0 ± 0.4
**3**	0.7 ± 0.3	0.8 ± 0.2	1.0 ± 0.3	0.6 ± 0.2
FmycT-K $^+$				
**1**	1.9 ± 0.2	5.2 ± 0.1	10.5 ± 0.4	15.5 ± 0.8
**2**	2.3 ± 0.2	3.8 ± 0.2	8.0 ± 0.08	11.9 ± 0.05
**3**	0.4 ± 0.4	0.1 ± 0.07	0.1 ± 0.03	1.5 ± 0.5
F10T-K $^+$				
**1**	0.1 ± 0.03	0.2 ± 0.1	0.4 ± 0.1	0.8 ± 0.01
**2**	0.2 ± 0.1	0.3 ± 0.1	0.2 ± 0.1	0.3± 0.01
**3**	0.02 ± 0.02	0.3 ± 0.05	0.4 ± 0.03	0.4 ± 0.1

3. Determination of apparent association constants by UV-Visible spectroscopy

Apparent association constants for ligands **1-3** were determined through UV-visible spectroscopy titration experiments. UV spectra were recorded on a Thermo Scientific BIOMATE 3S UV-vis Visible Spectrophotometer at ambient temperature. Measurements were taken in a 3 mL quartz cuvette with a path length of 10 mm. The UV-visible spectra were recorded between 600 nm and 300 nm and baseline corrected for the buffer used. The concentration of ligand was fixed at 10 µM in a constant volume of 1.5 mL buffer. The oligonucleotide sequence used was *EBR1* (5′-GGGCAGGGGGTGATGGGGAGGAGCCAGGG-3′). The oligonucleotide was purchased from Eurogentec (Belgium), purified by HPLC and delivered dry. Oligonucleotide concentration was determined by UV-absorbance using a NanoDrop 2000 Spectrophotometer from Thermo Scientific. The buffer used was 100 mM potassium phosphate pH 7.4. During the titration, aliquots of sample were removed and replaced with aliquots of oligonucleotide to give the required titration points (from a 100 µM stock solution in appropriate buffer containing also 10 µM ligand to maintain constant ligand concentration). NB: the oligonucleotide solution was annealed by heating to 90 °C for 2 minutes and then cooling on ice **prior** to the addition of ligand (to avoid annealing in the presence of ligand). Following addition, the solution was mixed

thoroughly and the UV-visible spectrum was acquired immediately. Data were fitted to an independent-and-equivalent-sites binding model (Equation 1) using Prism 7 software, a full derivation of which is provided by (amongst others) Thordarson,[3] adapted to an independent and equivalent sites model by (amongst others) Buurma and Gade.[4] The stoichiometry of the complex (*N*) was chosen as the lowest integer value that provided a satisfactory fit, $R^2 > 0.97$ (N = 2 for **1-3**). The data presented in Figure 2A shows the average values obtained from two independent experiments.

Equation 1:

$$\Delta A = \varepsilon_{\Delta complex}([complex])$$

where:

$$[complex] = \frac{1 + N \cdot K_a \cdot [DNA]_{tot} + K_a \cdot [ligand]_{tot} - \sqrt{(1 + N \cdot K_a \cdot [DNA]_{tot} + K_a \cdot [ligand]_{tot})^2 - 4 \cdot N \cdot K_a^{\,2} \cdot [D\text{[illegible]}}}{2 \cdot K_a}$$

$\Delta A = absorbance\ change\ at\ each\ titration\ point\ relative\ to\ free\ ligand\ (observed\ parameter)$

$\varepsilon_{\Delta complex} = change\ in\ the\ molar\ extinction\ coefficient\ between\ free\ ligand\ and\ DNA/ligand\ complex$

$N = the\ binding\ stoichiometery\ of\ ligand\ to\ DNA\ (selected\ parameter)$

$K_a = the\ apparent\ association\ constant$ (fitted parameter), $K_d = \frac{1}{K_a}$

$[DNA]_{tot} = the\ concentration\ of\ added\ DNA\ (known\ parameter)$

$[ligand]_{tot} = the\ total\ ligand\ concentration\ (fixed\ parameter)$

---

4. Circular dichroism titrations

Circular Dichroism (CD) titrations were recorded using a Jasco J-815 spectrometer fitted with a Peltier temperature controller. Measurements were taken in a quartz cuvette with a path length of 5 mm, at 20 °C, at a 1000 nm/min scanning speed at 1 nm intervals, with a 1 nm bandwidth. The CD spectra were recorded between 320 and 220 nm, and baseline corrected for the buffer used. The oligonucleotide sequence used was *EBR1* (5′-GGGCAGGGGGTGATGGGGAGGAGCCAGGG-3′). The oligonucleotide was purchased from Eurogentec (Belgium), purified by HPLC and delivered dry. Oligonucleotide concentration was determined by UV-absorbance using a NanoDrop 2000 Spectrophotometer from Thermo Scientific. The oligonucleotide was annealed before use by heating for 2 minutes at 90°C and then placed immediately into ice. The oligonucleotide was at a concentration of 5 µM which gave an OD of 1 and the buffer used was 100 mM pH 7.4 KPhos. The ligand was added by aliquot from a 1mM stock solution in the appropriate buffer (containing 10% DMSO to ensure solubility). The reported spectrum for each sample represents the average of 3 scans. Data processing was carried out using Prism 7 with an 8-point second order smoothing polynomial applied to all

spectra. Observed ellipticities were converted to mean residue ellipticity (θ) = deg cm^2 $dmol^{-1}$ (molar ellipticity).

5. Biological activity

Materials: *T. brucei* and MRC-5 cell lines used in the study are parto f the Instituto de Parasitologia y Biomedicina Lopez Neira collection and were originally purchased from ATCC.org

Parasite and cell culturing

T. brucei (Lister 427, antigenic type MiTat 1.2, clone 221a, bloodstream forms, "single marker" S427 (S16)) were cultured at 37 °C, 5% CO_2 in HMI-9 medium supplemented with 10% heat-inactivated fetal bovine serum (hiFBS, Invitrogen). *L. major* (MHOM/IL/80/Friedlin) promastigotes were cultured at 28 °C, 5% CO_2 in modified RPMI-1640 medium (Invitrogen, Carlsbad, CA) supplemented with 10% hiFBS. Parasites were maintained in culture in their experimental growth phase (below 2 million parasites per mL for *T. brucei* and 10 million parasites per mL for *L. major*), as previously described.[5]

MRC-5 cell line (human lung fibroblast) was grown in monolayer at 37 °C, 5% CO_2 in DMEM medium (1 g/L glucose) supplemented with 10% hiFBS, 100 U/mL penicillin, 100 mg/mL streptomycin and 2 mM L-glutamine.

Antiparasitic activity

The antitrypanosomal activity of the azobencene derivatives was determined by the alamarBlue® assay (ThermoFisher scientific).[6] Briefly, the stock solutions of the compounds were prepared in DMSO and the final DMSO percentage in each well was adjusted to be less than 1%. 1×10^4 parasites per mL were incubated at 37 °C, 5% CO_2 in 96-wells plates (100 µL/well) alone or in the presence of increasing concentration of compounds for 72 h. 20 µL of resazurin 1X solution (0,11 mg/mL) were then added to each well and the parasites were incubated for 4 h at 37 °C. Finally, cells were lysed with 50 µL per well of SDS 3%. The plate was incubated at 37 °C for an extra hour and then, fluorescence intensity was measured with an Infinite F200 plate reader (Tecan Austria, GmbH, Grödig, Austria), exciting at 550 nm and recording the emission at 590 nm. The results are expressed as the concentration of compound that reduces cell growth by 50% versus untreated control cells (IC_{50}). Data are presented as the average of at least three independent measurements all conducted in triplicate conditions.

The antileishmanial activity of the azobencene derivatives was measured using an MTT-based assay (Sigma-Aldrich, St. Louis, MO, USA).[7] The stock solutions of the compounds were prepared in DMSO and the final DMSO percentage in each well was adjusted to be less than 1%. 2×10^6 parasites per mL were incubated at 28 °C in 96-well plates (100 µL/well) alone or in the

presence of increasing concentration of compounds DMSO for 72 h. 10 µL of MTT (5 mg/ml) were added to each well and parasites were incubated for an extra 4 h at 28 °C. Finally, cells were lysed with 50 µL of 20% SDS per well. The plate was then incubated at 37 °C for an extra hour and then absorbance was measured at the Infinite F200 plate reader (TECAN Austria, GmbH, Grödig, Austria) at a wavelength of 540 nm. The IC_{50} was calculated as described above. Data are presented as the average of at least three independent measurements all conducted in triplicate conditions.

Cytoxicity

Cytotoxicity was measured in MRC-5 using the same alamarBlue® assay protocol described above for Trypanosoma Brucei. The only differences between the two protocols is the number of cells per well (5×10^3 cells in 100 µL per well in the case of MRC-5) and the necessity of an extra 24 h incubation period at 37 °C (5% CO_2) of the MRC-5 cells before compound addition, to allow the cells to attach and to stabilize.

6. References

[1] Y. Takebayashi, J. Ramos-Soriano, Y. J. Jiang, J. Samphire, E. Belmonte-Reche, M. P. O'Hagan, C. Gurr, K. J. Heesom, P. A. Lewis, T. Samernate, P. Nonejuie, J. Spencer, M. C. Galan, *bioRxiv* **2024**, 2022.2009.2001.506212.

[2] A. De Cian, L. Guittat, M. Kaiser, B. Sacca, S. Amrane, A. Bourdoncle, P. Alberti, M. P. Teulade-Fichou, L. Lacroix, J. L. Mergny, *Methods* **2007**, *42*, 183-195.

[3] P. Thordarson, *Chem Soc Rev* **2011**, *40*, 1305-1323.

[4] L. Hahn, N. J. Buurma, L. H. Gade, *Chemistry – A European Journal* **2016**, *22*, 6314-6322.

[5] M. Cabello-Donayre, S. Malagarie-Cazenave, J. Campos-Salinas, F. J. Gálvez, A. Rodríguez-Martínez, E. Pineda-Molina, L. M. Orrego, M. Martínez-García, M. P. Sánchez-Cañete, A. M. Estévez, J. M. Pérez-Victoria, *Molecular Microbiology* **2016**, *101*, 895-908.

[6] E. Belmonte-Reche, M. Martinez-Garcia, A. Guedin, M. Zuffo, M. Arevalo-Ruiz, F. Doria, J. Campos-Salinas, M. Maynadier, J. J. Lopez-Rubio, M. Freccero, J. L. Mergny, J. M. Perez-Victoria, J. C. Morales, *J Med Chem* **2018**, *61*, 1231-1240.

[7] J. M. Pérez-Victoria, B. I. Bavchvarov, I. R. Torrecillas, M. Martínez-García, C. López-Martín, M. Campillo, S. Castanys, F. Gamarro, *Antimicrobial Agents and Chemotherapy* **2011**, *55*, 3838-3844.
